# Association between gout, hyperuricaemia and Parkinson’s disease risk: a cohort study in western Sweden (2001–2017)

**DOI:** 10.1093/rap/rkaf102

**Published:** 2025-09-01

**Authors:** Mats Dehlin, Filip Bergquist, Panagiota Drivelegka, Tatiana Zverkowa Sandström, Lennart T H Jacobsson

**Affiliations:** Department of Rheumatology and Inflammation Research, Sahlgrenska Academy, University of Gothenburg, Gothenburg, Sweden; Department of Pharmacology, Sahlgrenska Academy, University of Gothenburg, Gothenburg, Sweden; Department of Neurology, Sahlgrenska University Hospital, Gothenburg, Sweden; Department of Rheumatology and Inflammation Research, Sahlgrenska Academy, University of Gothenburg, Gothenburg, Sweden; Department of Rheumatology and Inflammation Research, Sahlgrenska Academy, University of Gothenburg, Gothenburg, Sweden; Department of Rheumatology and Inflammation Research, Sahlgrenska Academy, University of Gothenburg, Gothenburg, Sweden

**Keywords:** gout, Parkinson’s, urate, allopurinol, risk, Sweden

## Abstract

**Objectives:**

Neurodegenerative diseases including Parkinson’s disease (PD) have been found to be associated with gout and hyperuricaemia in multiple studies but with conflicting results. We set out to determine the incidence and relative risk of PD in all gout individuals in western Sweden from 2001 to 2016 compared with controls. Blood urate levels in gout patients with and without incident PD were compared.

**Methods:**

All individuals with a gout diagnosis between 2001 and 2016 were identified and matched to population non-gout controls; individuals with prevalent PD were excluded. PD incidence rates were compared between cases and controls. The effect of gout on the risk of PD was calculated using Cox regression. The plasma urate level was identified for all gout cases.

**Results:**

In the 42 260 gout cases (67% male) and 174 747 controls (65% male) the incidence rate for PD was 1.38 per 1000 person-years in gout cases compared with 1.73 in controls, producing a significant decreased incidence rate ratio of 0.80 (95% CI 0.72, 0.90; *P* < 0.0001). Cox regression also showed a significant decreased risk for PD in the gout patients [hazard ratio 0.77 (95% CI 0.69, 0.86), *P* < 0.0001]. Gout patients with incident PD had significantly lower plasma levels of urate compared with gout patients without incident PD.

**Conclusion:**

The findings in this study show that gout is inversely associated with PD and in gout patients urate levels were inversely associated with the risk of PD. If future research proves this association to be causal it would have clinical implications in lowering urate in gout subjects at high risk of PD.

Key messages

- Having gout is associated with a decreased risk of developing Parkinson’s.
- Higher urate levels are associated with a lower risk of developing Parkinson’s in gout patients.

## Introduction

Gout is the most common inflammatory joint disease, with a prevalence ranging from <1% to 6% [1]. Supersaturation of body tissues with urate (hyperuricaemia) leads to formation and deposition of monosodium crystals that give rise to a strong inflammatory response, a gout attack. Thus, in gout, urate exerts pro-inflammatory effects, but urate is also a major antioxidant in humans [2].

Neurodegenerative diseases have been found to be associated with gout and hyperuricaemia in multiple studies, but with conflicting results, and a decreased risk for Alzheimer’s dementia (AD) [3, 4] and Parkinson’s (PD) [5, 6], but also with conflicting results [5, 7–11].

PD is the second most common neurodegenerative disease, with an increasing prevalence in all regions of the world [12]. The incidence of PD is age dependent and although there is a lack of modern studies examining PD prevalence in Sweden, the prevalence was 1% in women >65 years of age and 1.4% in men >65 of age in a recent population-based Norwegian study [13]. A typical pathological finding in PD is the accumulation of misfolded a-synuclein found in intracytoplasmic inclusions, so-called Lewy bodies, which contributes to neurodegeneration [14]. Reactive oxygen species and oxidative stress are considered to contribute pathogenically to PD, and since urate is an important antioxidant in humans, it could have a protective effect [15]. Furthermore, metal ions are found in misfolded a-synuclein and the chelating properties of urate may reduce the accumulation of metal ions [15]. In support of a role for urate, its levels in cerebrospinal fluid in patients with PD have been shown to be significantly reduced [16]. However, a randomized clinical trial showed no clinical benefit in early PD of increasing urate levels in the blood [17]. Risk factors associated with an increased risk for PD include pesticides and head injury [18], while smoking, caffeine consumption, physical activity and the use of ibuprofen have, in epidemiological studies, been associated with a reduced risk of PD [18].

To better understand the possible role of urate in PD, we set out to determine the incidence and relative risk (RR) of PD in all gout individuals in western Sweden (incident and prevalent gout cases) from 2001 to 2016 compared with non-gout controls, in total and stratified by sex and age. Finally, we also compared the levels of urate in the blood of gout patients with and without incident PD. We hypothesized that individuals with gout have a reduced risk of developing PD and that gout patients developing PD have lower levels of urate than gout patients who do not develop the disease.

## Methods

### Study design

This is a prospective regional population-based cohort stud, comparing a cohort of incident gout patients identified in the Western Swedish Health Care Register (VEGA) with an individually matched general population cohort identified from the Swedish Population Register. Through the unique personal identification number of each Swedish resident, the subjects were linked to a number of national and regional registers.

### Data sources

#### VEGA

The register contains information about all healthcare contacts in in- and outpatient secondary care clinics and in primary care in the Western Swedish Health Care Region (VGR). The date of contact and primary and secondary diagnoses given by the treating physician according to the Swedish version of the International Classification of Diseases, Tenth Revision (ICD-10) are registered. VEGA was used to identify gout cases and to retrieve information about relevant comorbidities for cases and controls. For gout cases, the date of the first gout diagnosis was used as the index date/date of identification.

#### The Cause of Death Register

The Swedish Cause of Death Register contains information on all deaths of people registered in Sweden. We identified cases and controls who died during follow-up in this register.

#### The Swedish Prescribed Drug Register

All prescribed drugs and the date of dispensation by Swedish pharmacies are recorded in the Swedish Prescribed Drug Register. Prescriptions for allopurinol (ATC code M04AA01) and all NSAIDs (ATC code M01A) was retrieved from here. The register has been available since 1 July 2005.

#### Clinical chemistry database of VGR

We identified the first laboratory values for urate and creatinine in cases from 2000 until the study end.

#### Study population

Gout is typically diagnosed and treated in a primary care setting. All inhabitants, ≥18 years of age, in the VGR from 1 January 2001 to 31 December 2016 constituted the target population. We identified all individuals ≥18 years of age with a diagnosis of gout in the VEGA register in 2001–2016. Gout was defined by the presence of an ICD-10 code for gout (M10) registered at a visit to a physician in the VEGA database. Up to 5 controls per case matched by age, year and municipality at the year of gout diagnosis were identified in the Swedish Census Register. The VEGA database was then searched for a diagnosis of PD back to 1 January 2000 and individuals with a prior diagnosis of PD were excluded. All included cases thus have a period free from a PD diagnosis of ≥1 year before their index date. PD was defined by the presence of an ICD-10 code for PD (G20.9) registered at a physician visit in the VEGA database. Follow-up started at the index date (date of first identified gout diagnosis) and continued to the date of first diagnosis of PD, death, emigration from the VGR or the end of the study (31 December 2017), whichever occurred first.

#### Outcome

The outcomes were the incidence of PD in cases and controls between 2001 and 2017 in total and stratified by age and sex. As a second outcome, the levels of urate in the blood of gout patients with and without incident PD were compared.

#### Confounders

The following confounders were recorded in the year of the index date for cases and controls: sex, education and birth outside Sweden. The following comorbidities were considered as possible confounders and were identified in the VEGA database ≥1 year prior to the year of the index date: alcohol-related disorders, hypertension, ischaemic heart disease, heart failure, cerebrovascular disease, diabetes mellitus (DM), dyslipidaemia, obesity, chronic kidney disease (CKD) and chronic obstructive pulmonary disease (COPD; as a proxy for smoking). For definitions, see Supplementary Table S1, available at *Rheumatology Advances in Practice* online.

#### Statistics

All analyses were performed in SAS 9.4 (SAS, Cary, NC, USA) and R 4.0.3 (R Foundation for Statistical Computing, Vienna, Austria). Continuous data were compared with *t*-tests and categorical data with chi-squared tests. The incidence rate of PD was calculated per 1000 person-years under the assumption of a Poisson distribution. The 95% CIs of the incidence rate ratios (IRRs) were calculated under the assumption of normal distribution for the natural logarithm of the IRR. We used two models for adjustment in the Cox regression. Model 1 adjusted for age and sex at baseline and model 2 also included education, DM, obesity, CKD, cerebrovascular disease and COPD.

In the Cox regression analysis we adjusted for violation of the proportionality assumption (when present) by including significant time interactions in the final models. To examine the possible impact of a competing risk of death on our results, we used the Fine and Gray competing risk regression model.

Ethical approval for the study was granted by the Ethical Review Board of Gothenburg, Sweden (Dnr 148-18). The need for informed consent was waived, as the data were derived from administrative registers that do not require such consent.

## Results

**Figure 1.**
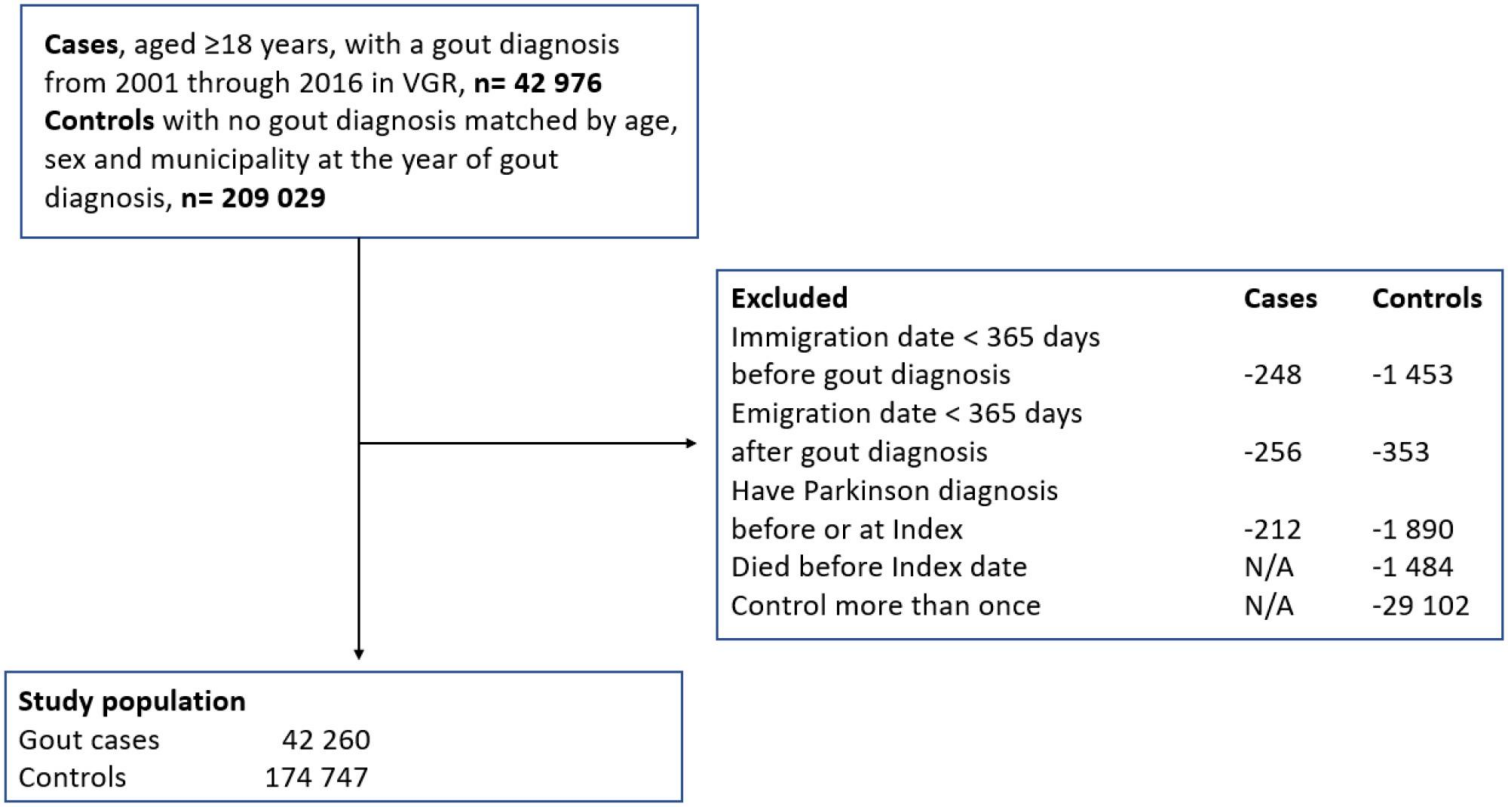
Flow chart of the study population. N/A: not applicable

After excluding individuals with prevalent PD we included 42 260 gout cases (67% male) and 174 747 controls (65% male) (Fig. 1, Table 1) in the study with a mean age of 68.4 years (s.d. 15.0) and 67.3 years (s.d. 15.2), respectively (Table 1). The gout patients had a significantly lower level of education, and all identified comorbidities were significantly more common in the gout cases compared with controls, including COPD (Table 1). Cases and controls had a median (Q1; Q3) follow-up time of 5.3 years [interquartile range (IQR) 2.6–8.6] and 5.5 years (IQR 2.8–8.8), respectively (Table 1). Interestingly, the proportion of cases excluded due to prevalent PD was significantly lower in the gout population (0.5%) compared with controls (1%) (*P* < 0.00001).

**Table 1.** Baseline characteristics of the study population.

Characteristics	Gout cases (*n* = 42 260)	Controls (*n* = 174 747)
Age, years, mean (s.d.)	68.4 (15.0)	67.3 (15.2)
Age, years, median (IQR)	70 (59–80)	69 (57–79)
Dead during follow-up, *n* (%)	15 180 (35.9)	49 007 (28.0)
Male, *n* (%)	28 418 (67.3)	114 324 (65.4)
Education, years, *n* (%)		
≤9	18 327 (43.4)	69 259 (39.6)
10–12	16 115 (38.1)	65 334 (37.4)
≥13	6967 (16.5)	36 572 (20.9)
Born outside Sweden, *n* (%)	5755 (13.6)	22 407 (12.8)
Comorbidities, *n* (%)		
Alcohol-related disorders	1228 (2.9)	2741 (1.6)
Hypertension	22 910 (54.2)	53 211 (30.5)
Ischaemic heart disease	9786 (23.2)	20 003 (11.4)
Heart failure	8221 (19.5)	10 486 (6.0)
Cerebrovascular disease	3951 (9.3)	11 161 (6.4)
DM	11 409 (27.0)	23 719 (13.6)
Dyslipidaemia	9380 (22.2)	20 951 (12.0)
Obesity	1709 (4.0)	2004 (1.1)
CKD	2254 (5.3)	1450 (0.8)
COPD	2915 (6.9)	6553 (3.7)
Follow-up time, years, median (IQR)	5.3 (2.6–8.6)	5.5 (2.8–8.8)

During follow-up, 353 (0.8%) incident cases of PD were identified in the gout cases compared with 1881 (1.1%) in the controls (*P* < 0.0001). The incidence rate for PD was 1.38 per 1000 person-years in gout cases compared with 1.73 in controls, showing a significant decreased IRR of 0.80 (95% CI 0.72, 0.90; *P* < 0.0001) for gout cases to develop PD compared with controls. Stratifying for sex gave a similar decrease in the IRR for male gout cases 0.82 (95% CI 0.72, 0.94) and nominally even lower for female gout cases [0.71 (95% CI 0.56, 0.90)] compared with controls. When stratifying for age, similar point estimates for IRR were seen in the older age group (>70 years), with an IRR for the gout cases in total of 0.74 (95% CI 0.64, 0.85), in men of 0.73 (95% CI 0.62, 0.85) and in women of 0.68 (95% CI 0.52, 0.90) (Table 2). In the younger age group (18–70 years), a decreased incidence was found for the total population of gout cases [IRR 0.80 (95% CI 0.66, 0.96)]; when stratified for sex the difference was not significant, but of the same direction in both sexes (Table 2). It should be noted that this group was considerably smaller compared with the older age group.

**Table 2.** Incidence of PD in cases and controls, in total and stratified by age and sex.

Population	*n* (%)		Person-years		IR per 1000 person-years		RR (95% CI)	*P*-value
	Cases	Controls	Cases	Controls	Cases	Controls		
Study								
All subjects	353 (0.84)	1881 (1.08)	254 933.3	1 089 132.0	1.38	1.73	0.80 (0.72, 0.90)	0.0001
Men	277 (0.97)	1377 (1.20)	177 216.9	724 716.1	1.56	1.90	0.82 (0.72, 0.94)	0.003
Women	76 (0.55)	504 (0.83)	77 716.4	364 415.9	0.98	1.40	0.71 (0.56, 0.90)	0.005
Age group 18–70 years								
All subjects	132 (0.62)	724 (0.77)	156 247.3	684 907.1	0.84	1.06	0.80 (0.66, 0.96)	0.02
Men	112 (0.69)	573 (0.82)	119 257.2	507 246.5	0.94	1.13	0.83 (0.68, 1.02)	0.07
Women	20 (0.39)	151 (0.63)	36 990.1	177 660.6	0.54	0.85	0.63 (0.40, 1.01)	0.055
Age group >70 years								
All subjects	221 (1.05)	1157 (1.43)	98 686.0	404 224.9	2.24	2.86	0.78 (0.68, 0.90)	0.0008
Men	165 (1.35)	804 (1.80)	57 959.7	217 469.6	2.85	3.70	0.77 (0.65, 0.91)	0.002
Women	56 (0.64)	353 (0.97)	40 726.3	186 755.3	1.37	1.89	0.73 (0.55, 0.96)	0.03

Cox regression also showed that the incidence of PD was significantly decreased in the gout cases, with an HR of 0.77 (95% CI 0.69, 0.86; *P* < 0.0001), when adjusting for age and sex and remained unchanged in the fully adjusted model 2 (Table 3). Stratified by sex, the inverse association of gout to incident PD was nominally higher in women with gout compared with men with gout in the fully adjusted model 2 [0.69 (95% CI 0.54, 0.88), *P* = 0.003] and 0.78 (95% CI 0.69, 0.89), *P* = 0.0002, respectively]. When stratifying for age, similar patterns were seen in the 18- to 69-year age group and in the >70-year age group in the fully adjusted model (Table 3). We performed a sensitivity analysis, where the possible impact of competing risks (death) on our results was evaluated with the method proposed by Fine and Gray, all point estimates were in the same direction as in the main analysis and of similar magnitude (Supplementary Table S2, available at *Rheumatology Advances in Practice* online).

**Table 3.** Time-dependent risk [HR (95% CI)] of incident PD during follow-up in cases compared with controls.

Study population	Overall		Male		Female	
	Model 1	Model 2	Model 1	Model 2	Model 1	Model 2
All subjects	0.77 (0.69, 0.86)	0.76 (0.68, 0.86)	0.78 (0.69, 0.89)	0.78 (0.69, 0.89)	0.71 (0.56, 0.90)	0.69 (0.54, 0.88)
Age group 18–70 years	0.74 (0.62, 0.90)	0.74 (0.61, 0.90)	0.76 (0.62, 0.94)	0.77 (0.63, 0.95)	0.64 (0.40, 1.02)	0.58 (0.36, 0.93)
Age group >70 years	0.76 (0.66, 0.88)	0.76 (0.66, 0.88)	0.77 (0.65, 0.91)	0.77 (0.65, 0.91)	0.72 0.55, 0.96)	0.74 (0.55, 0.98)

Model 1: adjusted for age and sex.

Model 2: adjusted for age, sex, education, DM, obesity, CKD, cerebrovascular disease, COPD.

Finally, we compared the first identified value of plasma urate and creatinine during follow-up in the gout cases with incident PD with the gout cases without. The gout patients with incident PD were older, more often male and had a higher mortality during follow-up compared with gout cases without PD: mean age 72.5 years (s.d. 9.2) years compared with 68.4 years (s.d. 15.0) (*P* < 0.0001), male sex 78.5% *vs* 67.2% (*P* < 0.0001) and death during follow-up 47.0% *vs* 35.8% (*P* < 0.0001) (Table 4). At baseline, there was no difference in the frequency of comorbidities except for heart failure, which was less common in the gout cases with PD (14.2% *vs* 19.5%; *P* = 0.01). During follow-up, a value of urate was retrieved in 65–67% and was found to be modestly but significantly lower in the gout cases with incident PD [mean 441.4 µmol/l (s.d. 119.2)] compared with those who were not diagnosed with PD [460.2 µmol/l (s.d. 132.2)] (*P* = 0.02). Furthermore, renal function–normalized plasma urate was calculated using the urate:creatinine ratio. Gout patients with incident PD had significantly lower urate:creatinine ratio at 4.1 compared with gout cases who were not diagnosed with PD [4.6 (*P* = 0.01)] (Table 4). A possible explanation for this could be differences in urate-lowering therapy (ULT) rates or dosages, but there was no difference in the frequency of allopurinol use within 125 days prior to the diagnosis of PD or study end between gout patients with incident PD and those who were not diagnosed with PD (35% *vs* 38%) and no difference in dose, with a mean dose of 139 mg (s.d. 80) in incident PD *vs* 141 mg (s.d. 80) in others (*P* = 0.9) (Table 4).

**Table 4.** Levels of urate, creatinine, demographics and comorbidities in gout patients with incident PD compared with gout patients without incident PD.

Characteristics	Gout patients with and without incident Parkinson’s		
	PD [*n* = 364 (0.9%)]	No PD [*n* = 41 896 (99.1%)]	*P*-value
Age, years, mean (s.d.)	72.5 (9.2)	68.4 (15.0)	<0.0001
Age, years, median (IQR)	73 (66–79)	70 (59–80)	<0.0001
Dead during follow-up, *n* (%)	166 (47.0)	15 014 (35.8)	<0.0001
Male, *n* (%)	277 (78.5)	28 141 (67.2)	<0.0001
Education (years), *n* (%)			
≤9	177 (50.1)	18 150 (43.3)	0.03
10–12	127 (36.0)	15 988 (38.2)	
≥13	46 (13.0)	6921 (16.5)	
Born outside Sweden, *n* (%)	44 (12.5)	5711 (13.6)	0.5
Comorbidities, *n* (%)			
Alcohol-related disorders	5 (1.4)	1223 (2.9)	0.09
Hypertension	193 (54.7)	22 717 (54.2)	0.9
Ischaemic heart disease	73 (20.7)	9713 (23.2)	0.3
Heart failure	50 (14.2)	8171 (19.5)	0.01
Cerebrovascular disease	39 (11.0)	3912 (9.3)	0.3
DM	75 (20.6)	11 334 (23.3)	0.3
Dyslipidaemia	79 (22.4)	9301 (22.2)	0.9
Obesity	9 (2.5)	1700 (4.1)	0.2
CKD	19 (5.4)	2235 (5.3)	0.97
COPD	16 (4.5)	2899 (6.9)	0.08
First plasma urate during follow-up			
Existing value, *n* (%)	237 (67.1)	27 409 (65.4)	0.5
Mean (s.d.), µmol/l	441.4 (119.2)	460.2 (132.2)	0.02
Median (IQR), µmol/l	433 (368; 509)	457 (376; 534)	0.02
First plasma creatinine during follow-up			
Existing value, *n* (%)	322 (88.5)	34 210 (81.6)	0.001
Mean (s.d.), mmol/mol	107.6 (58.7)	100.4 (56.2)	0.03
Median (IQR), mmol/mol	95 (81; 118)	90 (76; 108)	0.0001
First plasma urate/first creatinine during follow-up, mean (s.d.)	4.1 (1.2)	4.6 (1.7)	0.01
Medication 125 days before diagnosis of PD or study end			
Allopurinol, *n* (%)	112 (35.0)	15 383 (38.0)	0.3
Allopurinol daily dose, mg, mean (s.d.)	139 (80)	141 (80)	0.9
Allopurinol dose, mg, median (IQR)	100 (100–100)	100 (100–100)	0.9
NSAID, *n* (%)	47 (14.7)	3933 (9.7)	0.003
Follow-up time, years, median (IQR)	3.9 (1.6–6.8)	6.7 (3.9–10.1)	<0.0001

Regarding NSAIDs, there was a significantly larger proportion of the gout patients with incident PD who had a dispensed prescription of NSAIDs within 125 days prior to the diagnosis of PD/study end compared with those who were not diagnosed with PD (14.7 *vs* 9.7%; *P* = 0.003) (Table 4). However, a sensitivity analysis of the study population included between 2006 and 2016 (due to a lack of prescription data before 2006) that included prescription of NSAIDs within 125 days prior to the diagnosis of PD/study end as a confounder showed similar hazard rates as the main analysis (data not shown).

## Discussion

In this study we found a decreased risk of incident PD of ≈25% in patients with gout compared with non-gout controls; similar numbers were seen after stratifying for age and sex. Furthermore, gout patients with incident PD had significantly lower plasma levels of urate compared with gout patients without incident PD. In cases and controls excluded due to prevalent PD there were significantly fewer prevalent cases of PD among the gout patients compared with controls. This lends further observational evidence of an inverse association between urate levels and incident PD, although the observational design of the study does not preclude the possibility that urate is a marker of some other confounding protective factor that we have not been able to adjust for.

Mendelian randomization (MR) studies do not support a causal relationship between genetically determined high blood levels of urate and a risk of PD [7, 19, 20]. On the other hand, a meta-analysis by Chang *et al.* [5] addressing high urate levels and the risk of PD, which included eight studies comprising 91 307 participants and 1079 PD cases, found a decreased risk with increasing levels of serum urate, both in total and stratified by sex. The relative contribution of the most important risk factors for hyperuricaemia, diet, overweight and genes were investigated in the study by Topless *et al.* in 2021 [21]. In a gout cohort of 6781 patients, genetics accounted for 48% of the population-attributable fraction of hyperuricaemia while diet and weight contributed with 12% and 49%, respectively. Thus comorbidities and lifestyle factors substantially contribute to urate levels, offering a possible explanation for the negative findings from MR studies. The meta-analysis by Chang *et al.* [5] also addressed the risk of PD in patients with a gout diagnosis, based on seven studies comprising 358 509 gout patients and 13 608 PD cases. Slightly contradictory to the clear association between urate levels and PD, they found no association between gout and PD. They suggested this might be due to the use of ULT in the gout patients, rendering them less hyperuraemic.

The majority of studies on gout and the risk of PD have not shown any association [8, 9, 22–24] while the study by Pakpoour *et al.* [25] showed an increased risk of PD within 5 years after a hospitalisation for gout in England and the study by Singh and Cleveland [26] showed gout as an independent risk factor for PD in a 5% random sample of Medicare claims data, especially in the age group 65–75 years. In contrast, the study by De Vera *et al.* [27] found a decreased risk of PD in gout patients ≥65 years of age in Canada and Cortese *et al.* [28] found that prescription of ULT (irrespective of gout diagnosis) had a protective effect on PD development in the whole population of Norway. Differences in populations and outcome definitions may explain the contradictory findings.

If gout is protective against PD by effects of elevated urate, it can by hypothesised that gout patients who develop PD display lower urate levels than those who do not. Indeed, we found significantly lower blood levels of urate in the gout patients who developed PD, 441 µmol/l compared with those who did not, 460 µmol/l, and this was not obviously explained by the use of ULT since allopurinol was used by 35–38% in both groups and with similar mean doses. Although the difference is not clinically relevant in a gout context, it is consistent in direction with urate (or some other factor related to urate levels) influencing the risk of PD. In the study by Koros *et al.* [29] from 2023, a higher serum urate was found in patients with prodromal PD compared with clinical motor PD, possibly suggesting a protective effect of urate on the conversion to full-blown PD. However, the mean levels of urate in the study were well below the threshold for gout development. The SURE-PD3 trial (NCT02642393) investigated whether sustained urate-elevating treatment with inosine slows early PD progression [30]. This randomized clinical trial included 298 PD individuals, 49% women, with a mean age of 63 years, not yet requiring dopaminergic medication and with blood levels of urate <345 µmol/l randomized to receive inosine (*n* = 149) or placebo (*n* = 149). After 2 years of follow-up, no clinical benefit in disease progression was found between the groups. The participants taking inosine more frequently developed kidney stones compared with placebo, but no case of gout was reported, probably because of the modest increase in urate levels. The inosine group had a mean urate baseline level of 274 µmol/l, which increased to 393 µmol/l after 2 years, considerably lower compared with the gout patients in our study.

Oxidative processes have been implicated in several aspects of PD neurodegeneration, including mitochondrial dysfunction, dopamine metabolism, protein misfolding and inflammatory responses [31]. Early PD has also been associated with region-specific reduction in antioxidant-reduced glutathione [32, 33]. The hypothesis that urate has neuroprotective antioxidant effects stems from combining observations regarding associations between urate levels in neurodegenerative diseases and early observations that urate makes a significant contribution to the antioxidative capacity of the body. The antioxidant contribution of urate in blood has long been questioned [34] and, more importantly, its role in the CNS may be limited by low generation of urate in the CNS and poor blood–brain barrier permeability resulting in urate cerebrospinal fluid levels of ≈1/20 of those in blood [35]. One specific neuroprotective mechanism for urate may be inhibiting the breakdown of NF-E2-related factor 2 (Nrf2), since in dopamine neurons and astrocytes urate accumulates and promotes expression of Nrf2-regulated antioxidant genes and possibly other neuroprotective factors [36, 37]. Furthermore, PD may start in peripheral tissue, like the olfactory and enteric nervous systems [38], which means that peripheral urate may influence disease onset (but not necessarily progression) in spite of low CNS urate levels.

At which peripheral urate levels a neuroprotective effect can be expected is unknown—possibly at levels higher than in the SURE-PD3 trial. However, an influence of hyperuricaemia on the initiating events in PD pathology, but not disease progression, would be consistent with both our findings and the lack of effect on progression in the SURE-PD3 study. Along the same lines, at the time of diagnosis the disease may be too manifest to observe slowing of the degenerative process with a pharmacologically induced increase of urate. Not only are >50% of dopamine terminals degenerated [39], but the symptoms are also the result of plastic maladaptation that may progress in the dopamine-deficient brain [40]. In the meta-analysis by Chang *et al.* [5] of 91 307 participants and 1079 PD cases, the risk reduction increased with higher urate levels in a dose–response manner consistent with our findings. If future research proves this association to be causal, it would have clinical implications in lowering urate in gout subjects at high risk of PD.

There are some limitations to this study. First, in all register studies there is a risk for misclassification of the diagnosis. However, in a previous study we found that the gout diagnosis has high validity [41] and the same holds true for a PD diagnosis in Swedish registers [42]. Second, detection bias is a potential limitation to many register studies, including ours. The diagnosis of a chronic condition such as gout may have double-edged effects. Increased exposure to healthcare may lead to an increase in the diagnosis of other conditions but a chronic disease may also lead to a delay in the diagnosis of PD. Third, we miss data on some important lifestyle factors, including smoking, and the use of a COPD diagnosis as a proxy for it is only an approximation. Fourth, we lack detailed data on medication, which has been suggested to modify the risk of developing PD [43]. The use of NSAIDs has been identified as a possible protective factor, but a recent meta-analysis did not confirmed this [44]. Although NSAID use was expectedly higher among gout patients compared with controls, the use was unexpectedly also higher among gout patients who developed PD compared with those who did not. This, together with the unknown extent of non-prescription use of NSAIDs, makes current results difficult to interpret. Furthermore, reflecting gout management in Sweden, we have only retrieved urate plasma levels from two-thirds of the gout patients. Finally, our observations do not establish causality. As suggested by others, PD may in itself reduce urate due to changes in physical activity, reduced muscle mass and mitochondrial function [45]. There are also some strengths to this study. The study is population-based with a large sample size, minimizing the risk for selection bias, includes all patients with a gout diagnosis and has a long follow-up time. Data on comorbidities, cause of death and laboratory values were collected from three different registers that all have almost complete coverage of the population. Finally, sensitivity analyses accounting for competing causes for a PD diagnosis (death) showed similar results.

Our findings suggest that gout is inversely associated with PD, consistent across gender and age groups. Furthermore, in gout patients, higher urate levels are inversely associated with PD. The mechanisms behind this need further investigation.

## Supplementary Material

rkaf102_Supplementary_Data

## Data Availability

Data are available upon reasonable request through a strict controlled access procedure request to the corresponding author.
