# Supplementary material for: Association between gout, hyperuricaemia and Parkinson’s disease risk: a cohort study in western Sweden (2001–2017)
Source: Rheumatol Adv Pract. 2025 Sep 1;9(4):rkaf102. doi: 10.1093/rap/rkaf102 (PMC12496132; doi:10.1093/rap/rkaf102)
Supplement: rkaf102_Supplementary_Data [file rkaf102_supplementary_data.docx]

Supplementary Table S1: International Classification of Disease (ICD-10) codes for gout, Parkinson’s and comorbidities.

| **Diagnosis** | **ICD-10 code** |
| --- | --- |
| Alcohol related disorders | F10 |
| Cerebrovascular disease | I60–I69 |
| Chronic kidney disease | N18 |
| Chronic obstructive pulmonary disease | J44 |
| Diabetes mellitus | E10-14, O24 |
| Dyslipidemia | E78 |
| Gout | M10 |
| Hypertension | I10 |
| Heart failure | I50 |
| Ischemic heart disease | I20-I25 |
| Obesity | E66 |
| Parkinson’s | G20.9 |

Supplementary Table S2: Time-dependent risk, hazard ratio, of incident Parkinson’s during follow-up in cases compared to controls taking the possible impact of competing risk of death on our results by the Fine and Gray competing risk regression model.

| Study population |  |  |  |  |  |  |
| --- | --- | --- | --- | --- | --- | --- |
|  | Overall | | Male | | Female | |
|  | Model 1 | Model 2 | Model 1 | Model 2 | Model 1 | Model 2 |
| All subjects | 0.75  (0.67-0.84) | 0.74  (0.66-0.83) | 0.78  (0.69-0.89) | 0.77  (0.67-0.87) | 0.65  (0.51-0.82) | 0.64  (0.50-0.83) |
| Age group 18-70 | 0.77  (0.64-0.92) | 0.73  (0.61-0.88) | 0.80  (0.65-0.80) | 0.77  (0.63-0.95) | 0.61  (0.38-0.97) | 0.54  (0.33-0.88) |
| Age group 70+ | 0.72  (0.63-0.83) | 0.74  (0.64-0.85) | 0.73  (0.61-0.86) | 0.75  (0.64-0.89) | 0.65  (0.49-0.87) | 0.69  (0.52-0.93) |

Model 1: adjusted for age and sex.

Model 2: adjusted for age, sex, education, diabetes, obesity, chronic kidney disease, cerebrovascular disease, chronic obstructive pulmonary disease.
